# Membrane Lipid Microenvironment Modulates Thermodynamic Properties of the Na^+^-K^+^-ATPase in Branchial and Intestinal Epithelia in Euryhaline Fish *In vivo*

**DOI:** 10.3389/fphys.2016.00589

**Published:** 2016-12-15

**Authors:** Mario Díaz, Rosa Dópido, Tomás Gómez, Covadonga Rodríguez

**Affiliations:** Laboratorio de Fisiología Animal, Sección Biología, Departamento de Biología Animal, Facultad de Ciencias, Edafología y Geología, Universidad de La LagunaTenerife, Spain

**Keywords:** epithelial Na^+^-K^+^-ATPase, activation energy (*Ea)*, entropy of activation (Δ*H*^‡^), enthalpy of activation (Δ*S*^‡^), n-3 long chain polyunsaturated fatty acids (LCPUFA), DHA (docosahexaenoic acid), oleic acid, membrane lipid microenvironment

## Abstract

We have analyzed the effects of different native membrane lipid composition on the thermodynamic properties of the Na^+^-K^+^-ATPase in different epithelia from the gilthead seabream *Sparus aurata*. Thermodynamic parameters of activation for the Na^+^-K^+^-ATPase, as well as contents of lipid classes and fatty acids from polar lipids were determined for gill epithelia and enterocytes isolated from pyloric caeca, anterior intestine and posterior intestine. Arrhenius analyses of control animals revealed differences in thermal discontinuity values (*Td*) and activation energies determined at both sides of *Td* between intestinal and gill epithelia. Eyring plots disclosed important differences in enthalpy of activation (Δ*H*^‡^) and entropy of activation (Δ*S*^‡^) between enterocytes and branchial cells. Induction of n-3 LCPUFA deficiency dramatically altered membrane lipid composition in enterocytes, being the most dramatic changes the increase in 18:1n-9 (oleic acid) and the reduction of n-3 LCPUFA (mainly DHA, docosahexaenoic acid). Strikingly, branchial cells were much more resistant to diet-induced lipid alterations than enterocytes, indicating the existence of potent lipostatic mechanisms preserving membrane lipid matrix in gill epithelia. Paralleling lipid alterations, values of *Ea*_1_, Δ*H*^‡^ and Δ*S*^‡^ for the Na^+^-K^+^-ATPase were all increased, while *Td* values vanished, in LCPUFA deficient enterocytes. In turn, Differences in thermodynamic parameters were highly correlated with specific changes in fatty acids, but not with individual lipid classes including cholesterol *in vivo*. Thus, *Td* was positively related to 18:1n-9 and negatively to DHA. *Td, Ea*_1_ and Δ*H*^‡^ were exponentially related to DHA/18:1n-9 ratio. The exponential nature of these relationships highlights the strong impact of subtle changes in the contents of oleic acid and DHA in setting the thermodynamic properties of epithelial Na^+^-K^+^-ATPase *in vivo*. The effects are consistent with physical effects on the lipid membrane surrounding the enzyme as well as with direct interactions with the Na^+^-K^+^-ATPase.

## Introduction

In most epithelial tissues, the Na^+^-K^+^-ATPase is located at the basolateral membrane and provides the driving force for a variety of Na^+^-dependent transport processes across the plasma membrane, which are critical for sustaining animal homeostasis (Schuurmans-Stekhoven and Bonting, 1981; Skou and Esmann, 1992). In marine teleost fish, the coordinated physiology of gill and intestinal epithelia is responsible for the mobilization of ingested seawater toward the submucosal vascular beds, and the extrusion of the excess of NaCl from ingested seawater, leading to a net balance of water intake (Evans, 1998; Evans et al., 2005). The key molecular components in the machinery for the elimination of NaCl against a dramatic electrochemical gradient reside in a specialized cell type, namely chloride cells, which are located in the branchial epithelia (Evans, 1998; Evans et al., 2005). Despite the complexity of iono- and osmoregulatory processes in marine fish, involved mechanisms are ultimately dependent on the exergonic activity of the Na^+^-K^+^-ATPase (Evans, 1998; Evans et al., 2005).

Previous studies performed in our laboratory have disclosed differences in the biochemical properties of the Na^+^-K^+^-ATPase activities along the intestinal tract of the gilthead seabream, *Sparus aurata* (Díaz et al., 1998; Almansa et al., 2001; Dópido et al., 2004). On the basis of the differential sensitivities to ouabain, calcium and to ionic strength, it was concluded that α-subunit of Na^+^-K^+^-ATPase expression varied along the intestinal tract with α_1_ being ubiquitously expressed, but α_3_-subunit only observed in distal regions (Almansa et al., 2001). Later on, we found that differences in thermodynamic properties existed between intestinal segments from gilthead seabream and that these differences were likely due to different phospholipid microenvironment rather than to differential expression of α-subunits (Almansa et al., 2003). The influence of the lipid microenvironment composition on the thermodynamic and kinetic properties of the Na^+^-K^+^-ATPase is pervasive and has been recognized for long (Wheeler and Whittam, 1970; Klingenberg, 1975; Brasitus, 1983; Cornelius and Skou, 1984; Yeagle et al., 1988; Muriana et al., 1992; Gerbi et al., 1993, 1994; Ventrella et al., 1993). From these early studies it soon became clear that the degree of unsaturation of membrane phospholipids, as well as membrane cholesterol contents, played modulatory roles on the kinetic features of the Na^+^-K^+^-ATPase, which were initially interpreted as secondary to differences in membrane fluidity. However, the precise molecular association between degree of phospholipid unsaturation, type of phospholipid, cholesterol-to-phospholipid relationships, on the membrane biophysics (and geometry) and dynamics of membrane-bound integral proteins is just starting to be unraveled. Studies using membrane models have demonstrated direct interactions between Na^+^-K^+^-ATPase α-subunits and specific membrane phospholipids and fatty acids, and that there exit interaction sites at the hydrophobic surfaces of both α-subunits and regulatory FXYD protein in the buried helixes exposed to the phospholipid bilayer (Arora et al., 1998; Cohen et al., 2005; Esmann and Marsh, 2006). At present, there exist solid evidence for direct and specific interactions of different phospholipids and cholesterol which affect both the stability and molecular activity of the Na^+^-K^+^-ATPase, with essential roles in physiological regulation linked to membrane lipid composition (Gerbi et al., 1993, 1994; Yeagle et al., 1988; Crockett and Hazel, 1997; Else and Wu, 1999; Cornelius, 2001; Almansa et al., 2003; Esmann and Marsh, 2006; Cornelius et al., 2015). Unlike model membranes, where lipid composition is set to specific compositions, usually containing few molecular lipid species, membranes from living cells display an enormous biochemical complexity with ~10,000 different molecular species, likely depending on the organism and cell type, which, in turn, are subjected to continuous remodeling in response to intracellular and extracellular signals (Dowhan, 1997; Ernst et al., 2016). Important (and abundant) lipid molecules tightly associated to membrane physical properties in living cells are polyunsaturated fatty acids (LCPUFA). These fatty acids generally esterify glycerol backbone at *sn*-2 position in membrane phospholipids. Unlike saturates and monoenes, which can be synthesized by most vertebrates, including marine fish, LCPUFA cannot be produced (or are produced in very limited amounts) and their needs are mostly covered by their food intake. Amongst most important LCPUFA fatty acids, those belonging to the n-3 series (n-3 LCPUFA) and n-6 series (n-6 LCPUFA) are considered essential (Sargent et al., 1995; Díaz and Marín, 2013; Spector and Kim, 2015), and their deficiency are associated with a number of pathophysiological conditions and with the failure in adaptive responses (Bell et al., 1986; Gerbi et al., 1993, 1994, 1998; Sargent et al., 1995; Bogdanov et al., 2008; Russo, 2009; Díaz and Marín, 2013; Matsunari et al., 2013).

In the present study we have aimed to determine the influence of n-3 LCPUFA-deficient diets in the lipid composition of isolated intestinal and branchial epithelial cells of the gilthead seabream *in vivo*, and the extent to which such lipid modifications impact the thermodynamic properties of Na^+^-K^+^-ATPase from epithelial cells. The outcomes indicate that branchial epithelium is much more resistant to diet induced-LCPUFA depletion, in terms of phospholipid remodeling, than the intestinal counterparts. To the best of our knowledge, this is the first study demonstrating that the lipid composition of epithelial cells differs depending on their histological origin. Also, the results disclose severe changes in the thermodynamic behavior of the Na^+^-K^+^-ATPase that correlate with altered lipid environment-protein interactions *in vivo*.

## Materials and methods

### Animals, diets, and cell preparation

Gilthead seabream (*S. aurata*) (average weight 400 g) were initially reared at the National Institute of Oceanography of Tenerife (Spain) in seawater (35°/_oo_) at 20°C and fed commercial fish pellets (CONTROL), containing 2.07% n-3 LCPUFA (dry weight basis, DWB). A subset of specimens were then reared in the Atlantic Ocean (Los Gigantes, Southwest coast of Tenerife, Spain) in parallel tanks containing 10 individuals each, and fed once a day with an amount of pellets equivalent to 1% their biomass of the control diet (CONTROL) or a LCPUFA-deficient diet (DEFICIENT) for 6 months. The experimental diet containing no n-3 LCPUFA and very low amounts of n-3 PUFA was based on olive oil as lipid source, and obtained from Stirling University's Fish Nutrition facility (UK). Detailed composition of diets is shown in Table 1. After decapitation, intestinal segments were isolated from pyloric caeca and from the proximal and distal portions of the intestine as described previously (Almansa et al., 2001). For branchial epithelia, animals were first perfused through continuous ventricular injection of Ringer physiological solution to remove blood cells from gills. All tissues were rinsed in ice-cold Ringer solution, and submitted to epithelial cells isolation following the procedures described in detail in Dópido et al. (2004). Isolated cells were immediately placed in the homogenization solution containing 50 mM sucrose, 20 mM TRIS, 1 mM EDTA, 1 mM of the protease inhibitor, phenylmethylsulfonyl fluoride (PMSF), pH 7.5 (adjusted with TRIS/HCl) and kept at 4°C. Once homogenized, samples were stored in 1 ml aliquots at −80°C until analysis. All experimental manipulations were carried out following the procedures approved by the ethics committee (Comité de Ética de la Investigación y de Bienestar Animal: CEIBA) from Universidad de La Laguna.

**Table 1:** **Composition of control and PUFA-deficient diets**.

	**Control**	**Deficient**
Protein	40	38
Lipids	21	19
Celulose	3	5
Humidity	9	10
Ash	7	9
**Data in % Weight**		
**Fatty acids**		
	**Control**	**Deficient**
14:0	6.8	tr
16:0	18.9	11.8
16:1	7.6	1.4
18:0	3.7	2.9
18:1	12.7	72.1
18:2 n-6	4.3	9.6
18:3 n-3	1.4	0.6
18:4 n-3	2.9	–
20:1	2.4	0.4
20:2 n-6	0.2	tr
20:4 n-6	0.7	tr
20:4 n-3	0.7	tr
20:5 n-3	12.6	tr
22:1	2.2	0.4
22:5 n-6	0.4	tr
22:5 n-3	1.4	tr
22:6 n-3	13.2	tr
Monoenes	26.3	74.3
Saturates	30.7	14.7
n-3 PUFA	32.2	0.9
n-6 PUFA	5.9	9.8
n-3 LCPUFA	27.9	tr
n-6 LCPUFA	1.1	tr
n-3/n-6	5.5	0.1

*Data in % area. PUFA: polyunsaturated fatty acids (≥2 double bonds). LCPUFA: Long-chain polyunsaturated fatty acids (≥18 carbons and ≥2 double bonds)*.

### ATPase assays

Na^+^-K^+^-ATPase activities were measured in triplicate as the difference in inorganic phosphate (Pi) production from ATP in the presence or absence of 1 mM ouabain, under steady-state conditions, as described previously (Ventrella et al., 1990, 1993; Díaz et al., 1998; Almansa et al., 2001). Briefly, 25 μl of protein suspension, containing 50–100 μg protein, were added to test tubes containing 1 ml of incubation media (25 mM HEPES, 200 mM NaCl, 10 mM KCl, 5 mM MgCl2, and adjusted to pH 7.4 with TRIS) and allowed to preincubate for 5 min in a temperature-controlled water-jacketed chamber set at the desired temperature. The reaction was started by the addition of 50 μl (5 mM final concentration) of vanadate-free ATP and incubated for 10 min at different temperatures ranging from 4 to 50°C under continuous agitation. The amount of Pi produced was determined by the method of Forbush (1983). Accordingly, at the end of the incubation period, the reaction was stopped by adding 1 ml of an ice-cold solution containing 2.8% ascorbic acid, 0.48%, ammonium heptamolybdate, 2.8% SDS and 0.48 M HCl, and the tubes were placed at 4°C for 10 min. Afterwards, the reaction was developed by incorporating 1.5 ml of 2% sodium citrate, 2% sodium-m-arsenite and 2% acetic, incubated at 37°C for another 10 min, and the absorbance read at 705 nm. Na_2_HPO_4_ was used as Pi standard for calibration curves. Corrections for unspecific ATP hydrolysis were made by measuring the amount of Pi liberated in the absence of protein samples at each temperature tested. Specific Na^+^-K^+^-ATPase activities were expressed as μmol Pi/mg prot.hr.

### Lipid analysis

Gill and intestinal epithelial homogenates were also submitted to lipid analyses. For these purposes, cell collections were homogenized in chloroform/methanol (2:1 v/v) containing 0.01% of butylated hydroxytoluene as antioxidant, and then the organic solvent was evaporated under a stream of nitrogen. Lipid contents were determined gravimetrically. Lipid extracts were re-dissolved in chloroform/methanol (2:1 v/v) and stored in 1 mL glass vials in a nitrogen atmosphere free of O_2_ at −20°C until analyses.

Lipid classes were separated by one-dimensional double development high performance thin layer chromatography (HPTLC) as described elsewhere (Olsen and Henderson, 1989; Martín et al., 2006) and quantified by scanning densitometry using a Shimadzu CS-9001PC dual wavelength flying spot scanner. The polar lipid fraction (PL) was separated from neutral lipids by silica sep-pak cartridges and then subjected to acid-catalyzed transmethylation to yield fatty acid methyl esters (FAME) (Christie, 1982).

FAME were separated and quantified using a Shimadzu GC-14A gas chromatograph equipped with a flame ionization detector, an integrator and a fused silica capillary column Supelcowax TM 10 (30 m × 0.32 mm I.D.). Individual FAME were identified by reference to a multi-standard mixture which included authentic standards from marine fish (Supelco, Bellefonte, USA) and further confirmation of identity carried out by mass spectrometry when necessary.

### Statistics and calculations

All results are expressed as means ± SEM for, at least, four different determinations. Experimental data were submitted to one-way ANOVA followed by Tukey's test or to Kruskall-Wallis analysis followed by Mann-Withney *U* test, were appropriate. Correlation and determination coefficients were obtained by Pearson's approach. Multivariate analyses were performed using Principal Component Analyses (PCA). Statistical calculations were performed using SPSS (v.15.0 SPSS Inc., Chicago). Estimations of regression equations (lineal and exponential) and related parameters were performed by non-linear regression analysis tools using *Sigma Plot* software (Jandel Scientific, San Rafael, CA). A *p*-value below 0.05 was considered to achieve statistical significance.

### Materials

Ouabain, sodium dithionite, vanadate-free Na_2_ATP, HEPES, EDTA, TRIS, type IV Collagenase and PMSF were purchased from Sigma-Aldrich (Biosigma, Spain). Dimethylsulfoxide and HPTLC plates were obtained from Merck (Germany). Silica sep-pak cartridges were supplied by Millipore (Milford, MA). All reagents were analytical grade.

## Results

### Lipid profiles in isolated gill and intestinal epithelial cells from control fish

Lipid composition of gill epithelia and isolated enterocytes obtained from animals fed control diets are shown in Table 2. It can be observed that neutral and polar total lipid contents as well as individual lipid classes are rather homogeneous between intestinal segments. Only phospahtidylglicerol (PG, lowest in posterior intestine) and free fatty acids (FFA, lowest in anterior intestine) significantly differ between regions. However, it turns out that lipid profiles in epithelial cells from gill origin notably differ from those of enterocytes. Thus, gill epithelial cells contain lower amounts of phosphatidylcholine (PC), phosphatidylinositol (PI), phospahtidylglycerol (PG), phosphatidylserine (PS), phosphatidylethanolamine (PE), and as a consequence, total polar lipids (TPL), as well as higher levels of cholesterol (CHO), sterol esters (SE), free fatty acids (FFA) and resultant total neutral lipids (TNL) compared to enterocytes (Table 2).

**Table 2:** **Lipid classes and total polar lipid fatty acid composition of isolated epithelia from gilthead seabream reared under standard conditions**.

	**Pyloric caeca**	**Anterior intestine**	**Posterior intestine**	**Gill epithelia**
**LIPID CLASSES**				
LPC	0.27 ± 0.46	0.18 ± 0.21	0.27 ± 0.65	0.20 ± 0.29
SM	1.85 ± 0.56	2.08 ± 0.70	2.10 ± 0.86	1.04 ± 0.44
PC	19.38 ± 2.46a	17.49 ± 2.13a	17.77 ± 1.87a	4.21 ± 1.50b
PS	3.36 ± 0.80a	3.23 ± 0.56a	2.81 ± 0.89ab	1.08 ± 0.46b
PI	4.66 ± 0.57a	4.30 ± 0.77a	3.72 ± 0.70a	1.03 ± 0.21b
PG	5.31 ± 0.58a	4.31 ± 0.95a	3.11 ± 0.78b	1.02 ± 0.77c
PE	12.13 ± 2.14a	10.97 ± 1.86a	10.27 ± 2.54a	3.03 ± 2.10b
TPL	46.96 ± 6.48a	42.56 ± 5.46a	42.02 ± 6.56a	11.61 ± 4.91b
MAG	1.93 ± 1.14b	1.92 ± 1.12b	1.41 ± 1.24b	7.39 ± 3.82a
DAG	0.00 ± 0.00	0.00 ± 0.00	0.27 ± 0.67	0.52 ± 0.74
CHO	18.95 ± 2.24b	18.42 ± 1.64b	19.46 ± 0.57b	25.83 ± 2.11a
FFA	1.78 ± 1.83b	5.33 ± 3.64a	7.39 ± 4.53a	14.44 ± 6.08a
TAG	23.24 ± 5.16b	24.48 ± 2.50b	23.16 ± 4.14b	33.46 ± 1.24a
SE	5.57 ± 1.66	5.61 ± 2.81	6.06 ± 2.00	3.22 ± 0.36
TNL	53.04 ± 6.48b	57.44 ± 5.46b	57.98 ± 6.56b	84.86 ± 6.72a
LT (μg/mgprot)	554 ± 60a	375 ± 49b	345 ± 53b	560 ± 80a
**FATTY ACIDS**				
14:0	1.07 ± 0.53b	0.44 ± 0.24b	0.32 ± 0.21b	2.05 ± 0.11a
16:0	21.99 ± 1.38	21.34 ± 1.09	21.05 ± 0.92	22.08 ± 0.75
16:1	0.77 ± 0.34b	0.45 ± 0.17b	0.57 ± 0.22b	3.73 ± 0.64a
18:0	10.31 ± 0.79b	11.04 ± 0.84ab	12.11 ± 0.23a	8.56 ± 0.45b
18:1 n-9	9.84 ± 0.62b	10.70 ± 0.27b	10.10 ± 0.87b	14.85 ± 0.21a
18:2 n-6	2.49 ± 0.05	2.49 ± 0.03	3.21 ± 0.40	2.85 ± 0.01
18:3 n-3	0.00 ± 0.00b	0.00 ± 0.00b	0.00 ± 0.00b	0.17 ± 0.00a
18:4 n-3	0.09 ± 0.08	0.12 ± 0.10	0.13 ± 0.11	0.17 ± 0.02
20:1	1.41 ± 0.14b	1.98 ± 0.40b	1.49 ± 0.13b	0.45 ± 0.16a
20:2 n-6	0.36 ± 0.07	0.30 ± 0.03	0.20 ± 0.23	0.00 ± 0.00
20:4 n-6	2.59 ± 0.27b	2.41 ± 0.18b	2.62 ± 0.25b	5.10 ± 0.43a
20:4 n-3	0.48 ± 0.04a	0.53 ± 0.06a	0.54 ± 0.03a	0.39 ± 0.05b
20:5 n-3	6.15 ± 1.55b	6.32 ± 1.94b	6.14 ± 1.13b	9.15 ± 0.38a
22:1	0.25 ± 0.05a	0.40 ± 0.27a	0.30 ± 0.16a	0.00 ± 0.00b
22:5 n-6	0.46 ± 0.04	0.56 ± 0.07	0.60 ± 0.05	0.57 ± 0.10
22:5 n-3	2.25 ± 0.36a	2.47 ± 0.23a	2.55 ± 0.21	2.53 ± 0.32
22:6 n-3	34.13 ± 2.17a	35.04 ± 0.79a	32.70 ± 1.50a	21.69 ± 0.33b
**TOTALS AND INDEXES**				
Monoenes	12.29 ± 0.90b	13.53 ± 0.53b	12.47 ± 0.58b	20.19 ± 0.21a
Saturates	33.69 ± 1.71	33.35 ± 1.72	34.03 ± 1.14	34.06 ± 0.67
n-6	6.03 ± 0.38b	5.98 ± 0.09b	6.86 ± 0.53b	8.85 ± 0.57a
n-3 LCPUFA	43.03 ± 1.93a	44.38 ± 1.67a	41.95 ± 1.33a	33.76 ± 1.08b
18:1/n-3 LCPUFA	0.16 ± 0.00b	0.17 ± 0.01b	0.17 ± 0.01b	0.44 ± 0.01a
UI	248.96	256.74	244.40	194.35

*Different letters in the same row state for statistical differences with p-values below 0.05. LPC, Lyso-phosphatidylcholine; SM, Sphingolipids; PC, Phosphatidylcholine; PS, Phosphatidylserine; PI, Phosphatidylinositol; PG, Phosphatidylglycerol; PE, Phosphatidylethanolamine; TPL, Total Polar Lipids; MAG, Monoacylcerides; DAG, Diacylcerides; CHO, Cholesterol; TAG, Triacylcerides; FFA, Free fatty acids; SE, Sterol esters; TNL, Total Neutral Lipids; TL, Total Lipids; UI, Unsaturation Index. With the exception of 18:1/n-3 LCPUFA ratio, UI and TL, all data are expressed as % of total lipid. TL is expressed as μg lipids/mg total protein*.

Regarding fatty acids from polar lipids, significant differences between enterocytes were observed for stearic acid (18:0), linoleic acid (18:2n-6) and docosapentaenoic acid (DPA, 22:5n-6), which were higher in posterior intestine. However, when compared with gill epithelium significant differences in most fatty acids were observed. Thus, levels of palmitoleic acid (16:1), linoleic acid (18:2n6), linolenic acid (18:3n-3), and total monoenoic and total n-6 fatty acids were higher than in enterocytes, while contents of eicosatetraenoic acid (20:4n-3), eicosapentaenoic acid (20:5n-3), docosahexaenoic acid (22:6n-3) and total n-3 LCPUFA were notably lower than in the enterocytes within the same animals. Consequently, the 18:1/n-3 LCPUFA relationship, was significantly higher in gill epithelial cells.

**Figure 1:**
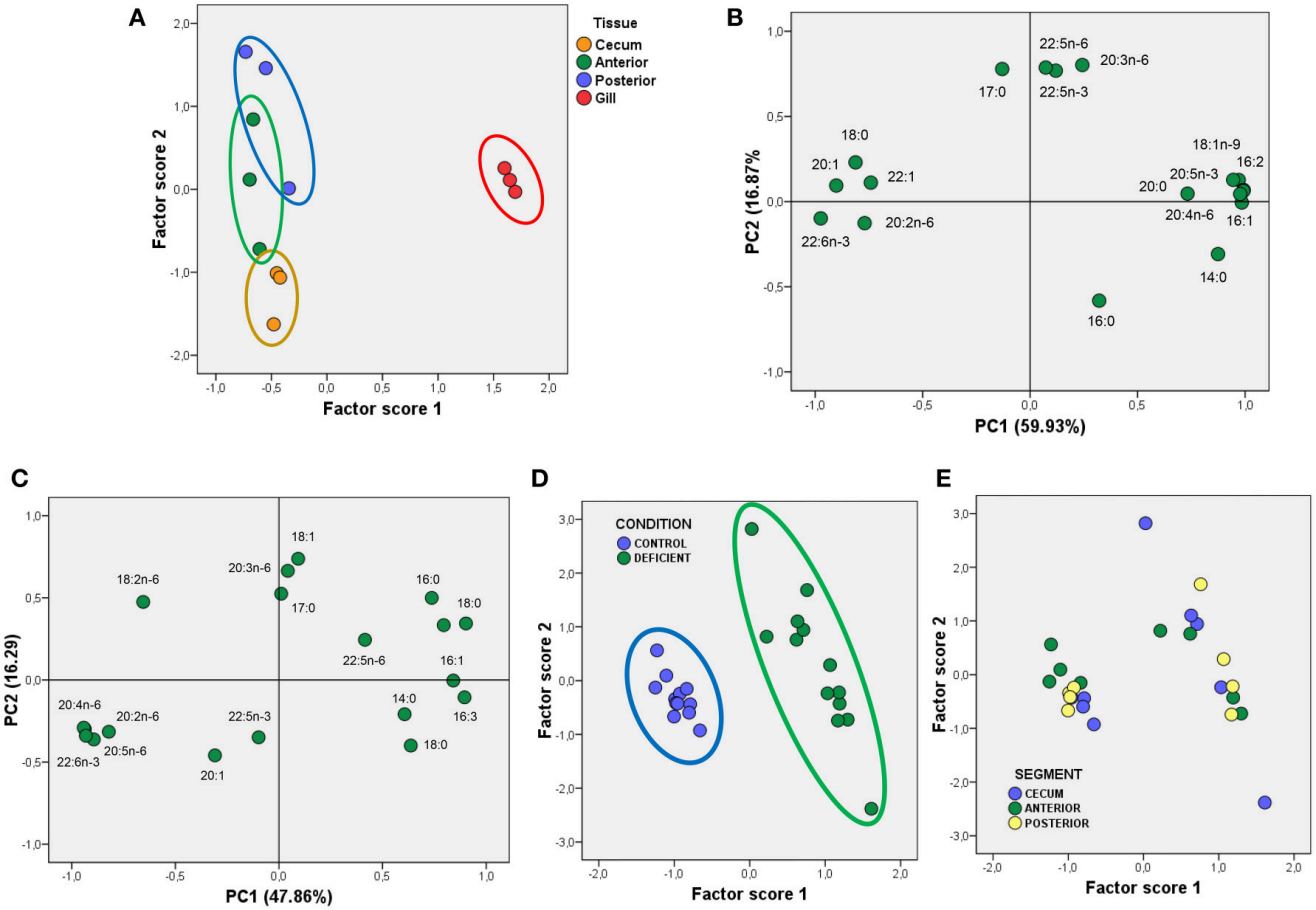
**Multivariate analyses using PCA of fatty acids from total polar lipids in gilthead seabream epithelia. (A)** Scatterplot for Factor scores 1 and 2, and grouped to identify different epithelial sources in control animals. **(B)** Factor loadings for Principal Components 1 and 2 (PC1 and PC2) obtained for fatty acids from TPL in control animals. The fraction of total variance explained by each principal component is indicated in parentheses. **(C)** Factor loadings for Principal Components 1 and 2 (PC1 and PC2) obtained for fatty acids from TPL in enterocytes from Control and LCPUFA-deficient animals. As in **(B)**, the fraction of total variance explained by each principal component is indicated in parentheses. **(D,E)** Scatterplots for Factor scores 1 and 2 grouped to identify different diets **(D)**, and enterocyte sources **(E)**. For details see corresponding results section.

The results presented so far reveal that lipid profiles in gill epithelia substantially differ from that in enterocytes. This was further confirmed by multivariate analyses using the principal component analyses (PCA). The results of the factor scores plot shown in Figure 1 indicate that the three populations of enterocytes were clustered together while the gill population was clearly segregated (Figure 1A). The variables explaining most variance (overall variance 76.90%) were 18:1n-9, 20:4n-6 (ARA, arachidonic acid), linolenic acid (18:3n-3), myristic acid (14:0), which were positively related to PC1, and DHA, stearic acid (18:0) and 20:1 which were negatively related to PC1 (Figure 1A). PC2 was positively correlated to three n-6 fatty acids (18:2n-6 or linoleic acid, 20:3n-6, and 22:5n-6) and negatively to palmitic acid (16:0). Indeed, differences in PC2, allowed a certain differentiation between enterocytes, with caecal and posterior enterocytes appearing as opposite clouds, and anterior enterocytes positioned between them (Figure 1B).

### Effects of n-3 PUFA-deficient diets on lipid profiles of isolated gill and intestinal epithelial cells

In the next experiment we aimed to determine the impact of dietary n-3 PUFA deficiency in the lipid composition of the four populations of epithelial cells. The results in Table 3 indicate that TPL were reduced by the treatment in enterocytes (at least in caecal and anterior enterocytes). This effect was due to general reductions of PC, PS, PI, and PE in deficient animals although differences only scarcely reached statistical significance. However, none of these trends were observed in gill epithelia. Regarding total neutral lipids, a generalized increase in the contents of SE, triacylglycerides (TAG) and TNL was observed in enterocytes from deficient animals irrespective of the intestinal origin. As in the case of TPL, none of these effects were observed in branchial cells.

**Table 3:** **Effects of experimental diets on lipid classes and total polar lipid fatty acid composition of isolated epithelia**.

	**Pyloric Caeca**		**Anterior Intestine**		**Posterior Intestine**		**Gill epithelia**	
	**Control**	**Deficient**	**Control**	**Deficient**	**Control**	**Deficient**	**Control**	**Deficient**
**LIPID CLASSES**								
LPC	0.00 ± 0.00	0.00 ± 0.00	0.00 ± 0.00	0.00 ± 0.00	0.00 ± 0.00	0.00 ± 0.00	1.86 ± 0.12	0.68 ± 0.29^*^
SM	1.18 ± 0.36	1.08 ± 1.01	0.67 ± 0.06	0.61 ± 0.14	0.80 ± 0.04	0.41 ± 0.21^*^	1.52 ± 0.29	1.02 ± 0.38
PC	16.21 ± 5.36	10.31 ± 2.75	16.61 ± 0.10	11.30 ± 1.89^*^	13.61 ± 3.50	11.40 ± 3.23	8.23 ± 0.70	8.71 ± 3.06
PS	2.12 ± 0.25	1.58 ± 0.31	2.24 ± 0.49	2.04 ± 0.10	3.07 ± 1.00	2.04 ± 0.34	3.16 ± 0.31	3.79 ± 0.38
PI	4.50 ± 1.18	3.32 ± 0.70	3.82 ± 0.12	2.85 ± 0.73	3.28 ± 0.64	3.36 ± 0.61	2.49 ± 0.24	2.13 ± 0.41
PG	5.06 ± 0.51	3.89 ± 0.67	4.59 ± 0.02	3.49 ± 1.05	2.27 ± 1.64	3.29 ± 0.65	2.54 ± 0.112	2.44 ± 0.23
PE	12.14 ± 1.57	7.43 ± 1.04^*^	13.12 ± 0.30	10.97 ± 1.93	12.39 ± 2.51	13.30 ± 1.42	6.81 ± 0.43	6.11 ± 1.80
TPL	41.22 ± 8.18	27.63 ± 4.20^*^	41.07 ± 0.21	31.28 ± 5.21^*^	35.44 ± 7.40	33.84 ± 5.02	26.64 ± 0.46	24.88 ± 5.57
MAG	1.10 ± 0.82	1.32 ± 1.01	0.56 ± 0.05	0.15 ± 0.11^*^	1.05 ± 0.43	0.69 ± 0.08	1.33 ± 0.44	1.38 ± 2.23
DAG	0.00 ± 0.00	0.00 ± 0.00	0.00 ± 0.00	0.02 ± 0.03	0.00 ± 0.00	0.00 ± 0.00	0.35 ± 0.34	0.28 ± 0.66
CHO	28.90 ± 3.38	28.25 ± 3.18	21.37 ± 0.53	20.77 ± 1.62	26.46 ± 1.72	28.45 ± 0.84	28.52 ± 1.02	27.38 ± 3.78
FFA	8.72 ± 5.87	5.26 ± 2.75	12.66 ± 1.35	10.20 ± 3.03	17.25 ± 8.64	9.22 ± 1.23	10.21 ± 0.79	10.72 ± 3.20
TAG	17.18 ± 5.57	31.89 ± 5.62^*^	21.06 ± 1.45	33.51 ± 3.00^*^	16.64 ± 2.21	23.79 ± 4.27^*^	21.78 ± 1.33	21.07 ± 3.65
SE	2.85 ± 0.08	5.62 ± 0.93^*^	2.44 ± 0.13	4.03 ± 0.83^*^	2.91 ± 1.41	3.82 ± 0.67	11.13 ± 0.31	11.29 ± 0.70
TNL	58.77 ± 8.18	72.36 ± 4.20^*^	58.10 ± 0.63	68.71 ± 5.21^*^	65.04 ± 7.29	65.98 ± 5.03	73.35 ± 0.46	72.12 ± 5.57
PHO/CHO	1.43	0.98	1.92	1.51	1.34	1.19	0.87	0.88
LT (μg/mgprot)	767 ± 90	1090 ± 65^*^	865 ± 60	1230 ± 180^*^	761 ± 90	998 ± 190^*^	520 ± 20	518 ± 70
**FATTY ACIDS**								
14:0	0.42 ± 0.12	0.60 ± 0.24	1.45 ± 1.45	0.44 ± 0.28	0.59 ± 0.08	0.41 ± 0.20	0.96 ± 0.39	0.68 ± 0.23
16:0	16.88 ± 0.55	14.59 ± 2.57	18.61 ± 5.12	12.10 ± 2.68	17.36 ± 1.19	11.63 ± 0.67^*^	22.85 ± 3.92	22.65 ± 5.23
16:1	1.60 ± 0.32	1.78 ± 0.24	2.19 ± 1.03	1.19 ± 0.48	1.61 ± 0.23	1.18 ± 0.23	3.76 ± 0.50	2.19 ± 0.16^*^
18:0	11.22 ± 1.25	9.88 ± 1.80	13.11 ± 0.64	10.63 ± 2.72	13.47 ± 1.18	10.15 ± 1.69^*^	11.81 ± 2.01	12.11 ± 4.14
18:1n-9	9.07 ± 2.85	21.41 ± 6.72^*^	9.98 ± 3.34	23.84 ± 9.39^*^	6.86 ± 1.52	22.92 ± 5.35^*^	14.29 ± 3.22	20.51 ± 4.55
18:1n-7	2.73 ± 0.17	2.71 ± 0.10	2.76 ± 0.11	2.72 ± 0.20	2.31 ± 0.22	2.60 ± 0.20	3.06 ± 0.19	2.74 ± 0.30
18:1n-5	0.10 ± 0.09	0.11 ± 0.10	0.00 ± 0.00	0.00 ± 0.00	0.06 ± 0.06	0.06 ± 0.11	0.00 ± 0.00	0.09 ± 0.16
18:2n-6	5.29 ± 0.28	7.43 ± 1.97	4.87 ± 0.78	8.32 ± 3.42	4.95 ± 0.68	7.37 ± 1.50	5.81 ± 0.58	6.87 ± 2.92
18:3n-3	0.32 ± 0.06	0.24 ± 0.04^*^	0.44 ± 0.07	0.34 ± 0.05^*^	0.30 ± 0.08	0.27 ± 0.07	0.38 ± 0.04	0.11 ± 0.18^*^
18:4n-3	0.16 ± 0.08	0.20 ± 0.17	0.17 ± 0.07	0.10 ± 0.11	0.07 ± 0.06	0.14 ± 0.02	0.06 ± 0.11	0.05 ± 0.09
20:1	0.72 ± 0.06	1.05 ± 0.17	0.99 ± 0.13	0.92 ± 0.80	0.77 ± 0.06	1.52 ± 0.28^*^	1.47 ± 0.65	1.23 ± 0.24
20:2n-6	0.47 ± 0.04	0.53 ± 0.14	0.43 ± 0.09	0.58 ± 0.24	0.20 ± 0.34	0.28 ± 0.49	0.49 ± 0.07	0.64 ± 0.16
20:4n-6	2.68 ± 0.48	1.33 ± 1.18	1.56 ± 1.36	1.48 ± 0.42	2.16 ± 0.15	1.52 ± 0.21^*^	2.11 ± 0.96	1.78 ± 0.62
20:4n-3	0.63 ± 0.04	0.54 ± 0.16	0.60 ± 0.26	0.50 ± 0.24	0.56 ± 0.18	0.25 ± 0.26	0.10 ± 0.17	0.08 ± 0.13
20:5n-3	7.71 ± 0.65	6.10 ± 3.00	6.62 ± 2.28	5.92 ± 2.75	4.84 ± 1.56	4.37 ± 1.96	2.88 ± 1.08	1.37 ± 1.20
22:1	0.05 ± 0.04	0.10 ± 0.09	0.09 ± 0.09	0.14 ± 0.14	0.02 ± 0.04	0.05 ± 0.08	0.41 ± 0.72	0.00 ± 0.00
22:5n-6	0.70 ± 0.05	0.46 ± 0.15	0.79 ± 0.11	0.68 ± 0.40	0.96 ± 0.10	0.57 ± 0.22^*^	0.55 ± 0.14	0.24 ± 0.21^*^
22:5n-3	3.08 ± 0.50	2.32 ± 0.54	2.31 ± 0.74	2.26 ± 0.54	3.19 ± 0.51	2.50 ± 0.53	1.31 ± 0.32	1.23 ± 0.22
22:6n-3	27.12 ± 1.09	18.18 ± 2.10^*^	23.57 ± 3.39	18.17 ± 3.07^*^	28.83 ± 1.50	21.72 ± 2.32^*^	13.46 ± 4.99	11.87 ± 2.96
**TOTALS AND INDEXES**								
Monoenes	15.73 ± 2.42	28.17 ± 6.45^*^	17.31 ± 2.67	29.86 ± 9.76^*^	12.93 ± 0.98	29.55 ± 5.13^*^	25.54 ± 5.32	29.79 ± 4.23
Saturates	30.02 ± 1.25	26.56 ± 4.66	35.17 ± 7.79	24.39 ± 6.12	33.11 ± 2.76	23.37 ± 2.60^*^	37.46 ± 6.44	36.58 ± 9.60
n-3	39.20 ± 0.45	27.74 ± 5.67^*^	33.88 ± 6.20	27.32 ± 6.13	38.05 ± 3.35	29.60 ± 5.06	18.19 ± 6.56	14.71 ± 3.83
n-6	9.46 ± 0.29	10.40 ± 1.24	7.81 ± 2.14	11.20 ± 3.05	8.60 ± 0.46	10.14 ± 1.54	9.16 ± 1.70	9.82 ± 3.82
n-3LCPUFA	38.61 ± 0.43	27.17 ± 5.62^*^	33.10 ± 6.27	26.85 ± 6.13	37.51 ± 3.31	28.92 ± 4.74	17.75 ± 6.49	14.55 ± 3.56
n-3/n-6	4.15 ± 0.17	2.73 ± 0.89	4.44 ± 0.56	2.70 ± 1.42	4.44 ± 0.51	3.02 ± 0.97	1.95 ± 0.34	1.56 ± 0.29
18:1/n-3LCPUFA	0.23 ± 0.07	0.85 ± 0.43	0.30 ± 0.08	0.98 ± 0.61	0.18 ± 0.03	0.83 ± 0.34^*^	0.88 ± 0.35	1.42 ± 0.19^*^
UI	261.62	206.09	227.69	209.64	252.62	220.28	152.06	137.98

* *p < 0.05 compared to controls. Abbreviations as in Table 2*.

Profiles of fatty acids in polar lipids shown in Table 3, also indicate severe changes in enterocytes from deficient animals. Thus, a dramatic reduction of DHA occurs in all enterocyte preparations, reflecting the composition of deficient diets. Furthermore, the depletion of DHA in enterocytes was accompanied by pervasive increase in oleic acid (18:1n-9), linoleic acid and total monoenoic fatty acids. Strikingly, none of these changes appear to affect gill epithelium, as only few minor fatty acids were significantly changed.

Given that main differences between enterocytes from LCPUFA deficient animals were observed for fatty acids, we next performed multivariate analyses using PCA. The results illustrated on Figure 1C revealed that two principal components explained 64.1% of overall variance, with PC1 being positively related to saturates (mainly 14:0, 16:0, and 18:0) and negatively to polyunsaturated fatty acids (mainly 20:4n-6, 20:5n-3, and 22:6n-3). Interestingly, PC2 was positively related to 18:1n-9, but PC1 was insensitive to this fatty acid, despite large differences between groups. Plotted factor scores revealed that deficiency-induced changes in fatty acids composition of phospholipids was so dramatic that two clusters corresponding to control and deficient enterocytes were plainly segregated (Figure 1D). Further, when factor scores were plotted for each intestinal segment, it was observed that irrespective of the intestinal origin, the three groups of enterocytes are represented in both CONTROL and DEFICIENT clouds (Figures 1D,E).

### Thermodynamic properties of Na^+^-K^+^-ATPase in isolated enterocytes and branchial cells

**Figure 2:**
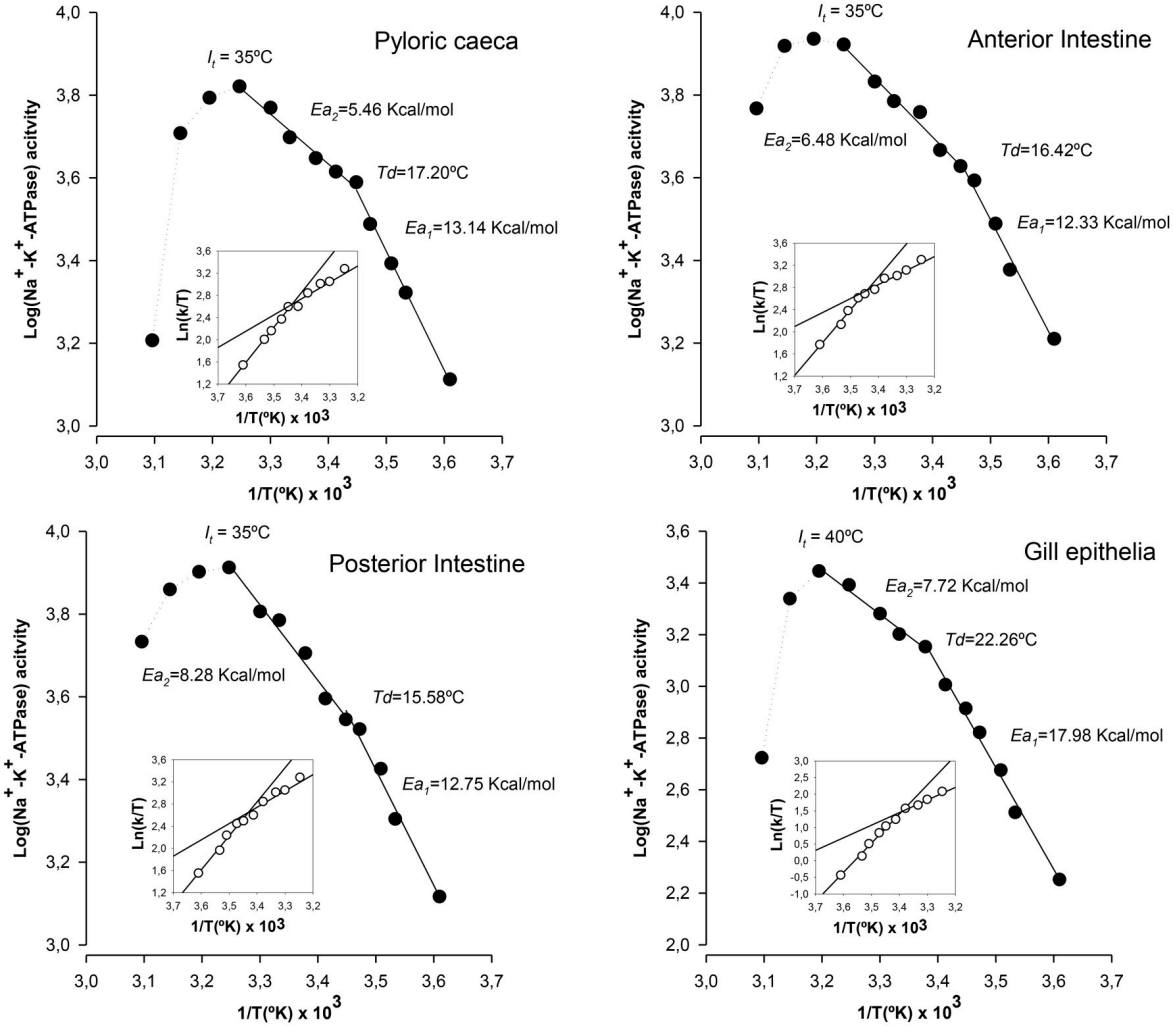
**Thermodynamic features of Na^**+**^-K^**+**^-ATPase from gilthead seabream epithelia reared under standard conditions**. Illustrated correspond to Arrhenius plots (Log[activity] vs. 1/T) and Eyring plots (insets, Ln[k/T] vs. 1/T) for the hydrolysis of ATP by Na^+^-K^+^-ATPase in each epithelial preparation. *Td*: Arrhenius breaking (or discontinuity) temperature. *Ea*_1_ and *Ea*_2_: activation energies below and above the point of discontinuity, respectively. *I*_*t*_: discontinuity at inactivation temperature. Each point corresponds to the average of four different experiments.

We next examined the temperature-dependence curves for the Na^+^-K^+^-ATPase reaction rates from the three groups of enterocytes and branchial cells under control conditions. Arrhenius plots for the Na^+^-K^+^-ATPase from intestinal and branchial preparations along with the Eyring plots (in the insets) are shown in Figure 2. Arrhenius plots indicate that the overall reactions of ATP hydrolysis for the Na^+^-K^+^-ATPase activities from all sources proceeded with temperature discontinuity points (*Td*) and two activation energies, i.e., above (*Ea*_1_) and below (*Ea*_2_) the breaking temperature. The calculated values for *Td* were lowest for posterior enterocytes (15.6°C) and highest for gill epithelia (22.3°C), being *Td* for pyloric caeca (17.2°C) and anterior intestine (16.4°C) enterocytes very close each together. Regarding *Ea*_1_ it is noticeable that values of enterocytes were similar between segments (in the range 12.33 kcal/mol – 15.58 kcal/mol), but notably lower than the value obtained in gill epithelia (17.98 kcal/mol). Conversely, *Ea*_2_ were similar between epithelial cells from intestine and gills (in the range 5.46 kcal/mol–7.72 kcal/mol). Of note, it can also be observed that maximal activities are attained in the range 30–35°C, well beyond the standard rearing (and environmental) temperature of this species, and that increasing the assay temperature above 45°C (*I*_*t*_, inactivation temperature) leads to a pronounced decrease in ATPase activity, which has been interpreted as result of progressive enzyme denaturation and/or destabilization of membrane microenvironment as thermal stress increases. This thermosensitive behavior of Na^+^-K^+^-ATPase is common between ectotherms (Else and Wu, 1999).

By applying the transition states theory using Eyring plots we estimated activation enthalpy (Δ*H*^‡^) and activation entropy (Δ*S*^‡^). Results are illustrated in the insets of Figure 2. From the slopes and intercepts of ln(k/T) vs. 10^3^/T plots below and above *Td*, it was calculated the activation enthalpy and entropy values. In general, lower enthalpy and entropy values are observed above *Td*. In enterocytes, Δ*H*^‡^ values were similar between sections and ranged between 11.78 kcal/mol and 12.50 kcal/ mol below *Td*, and between 4.92 kcal/mol and 7.77 kcal/mol above *Td*. These values were considerably higher for the case of gill epithelia, which were 18.53 kcal/mol and 10.06 kcal/mol as calculated below and above *Td*, respectively. Regarding activation entropy, highest entropy values were observed for gill epithelia both above (36.86 kcal/°K/mol) and below (65.95 kcal/°K/mol) *Td*, while in enterocytes, Δ*S*^‡^ remained similar and ranging between 48.49 kcal/°K/mol (caeca) and 46.01 kcal/°K/mol (anterior intestine) below *Td*, and 31.78 kcal/°K/mol (anterior intestine) and 22.07 kcal/°K/mol (caeca) above *Td*.

### Effects of alterations in lipid profiles on the thermodynamic properties of Na^+^-K^+^-ATPase

**Figure 3:**
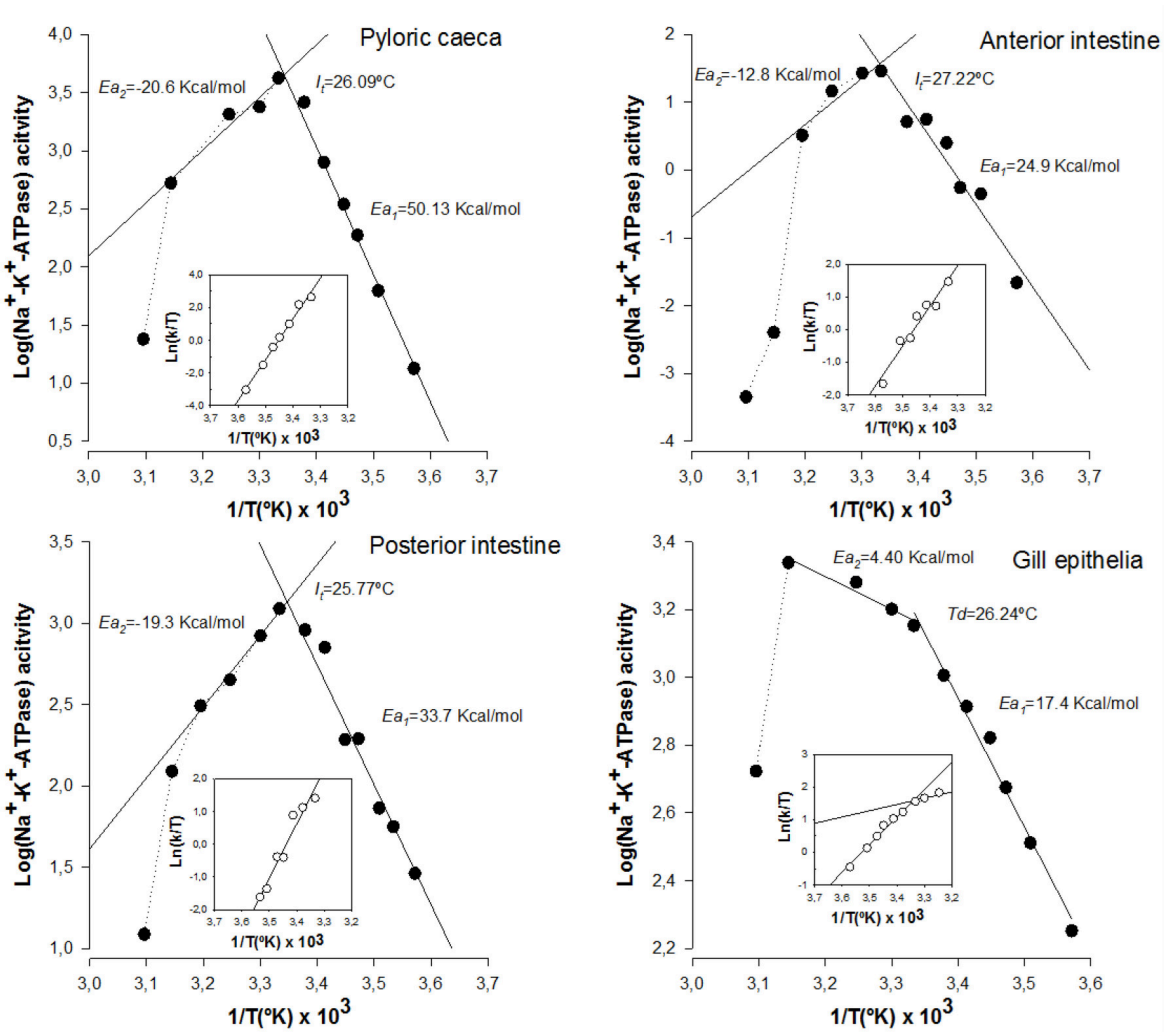
**Effects of PUFA-deficient diets on thermodynamic properties of Na^**+**^-K^**+**^-ATPase from gilthead seabream enterocytes from anterior intestine, posterior intestine, pyloric caeca, as well as from gill epithelium**. Illustrated correspond to Arrhenius and Eyring plots for the hydrolysis of ATP by Na^+^-K^+^-ATPase in each epithelial preparation as indicated in Figure 2. Each point corresponds to the average of four different experiments.

We next assessed the potential effects of lipid alterations induced by n-3 PUFA deficient diets on the thermodynamic properties of Na^+^-K^+^-ATPase from intestinal and branchial epithelial cells. The results are illustrated in Figure 3. The first obvious differences when compared to control cells were the disappearance of *Td* and the increase in activation energy values in the enzyme from enterocytes. Indeed, *Ea*_1_ values ranged from 50.13 kcal/mol in caecal enterocytes to 24.9 kcal/mol in the case of anterior enterocytes. The second most dramatic change was the increased thermosensitivity displayed by the enzyme from enterocytes, which started to inactivate (*I*_*t*_) around 26°C, nearly 20° lower than in control cells. Consequently, *Ea*_2_ could not be estimated in enterocyte populations. These deleterious effects of n-3 PUFA deficiency in enterocytes are indicative of membrane instability and protein inactivation as a consequence of environment destabilization. Strikingly, gill epithelium demonstrates, once again, a considerable resistance to LCPUFA depletion and its effects were much less prominent in *Td, Ea*_1_, and *Ea*_2_, though slight differences compared to control animals were detected for *Td* and *Ea*_2_, but not for *Ea*_1_.

Changes in activation enthalpy (Δ*H*^‡^) and activation entropy (Δ*S*^‡^) for Na^+^-K^+^-ATPase in enterocytes below (*I*_*t*_) were dramatic, with a generalized increase in Δ*H*^‡^ (24.36 kcal/mol, 33.14 kcal/mol and 44.61 kcal/mol in anterior, posterior and caecal enterocytes, respectively) and Δ*S*^‡^ (84.26 kcal/°K/mol, 113.94 kcal/°K/mol and 171.27 kcal/°K/mol in anterior, posterior and caecal enterocytes, respectively). However, for gill epithelia, Δ*H*^‡^ values remained similar to controls both above (10.06 kcal/mol) and below (18.53 kcal/mol) *Td*. Likewise, Δ*S*^‡^ changes closely resembled those of controls with values of 36.68 kcal/°K/mol and 65.95 kcal/°K/mol, above and below discontinuity point, respectively.

**Figure 4:**
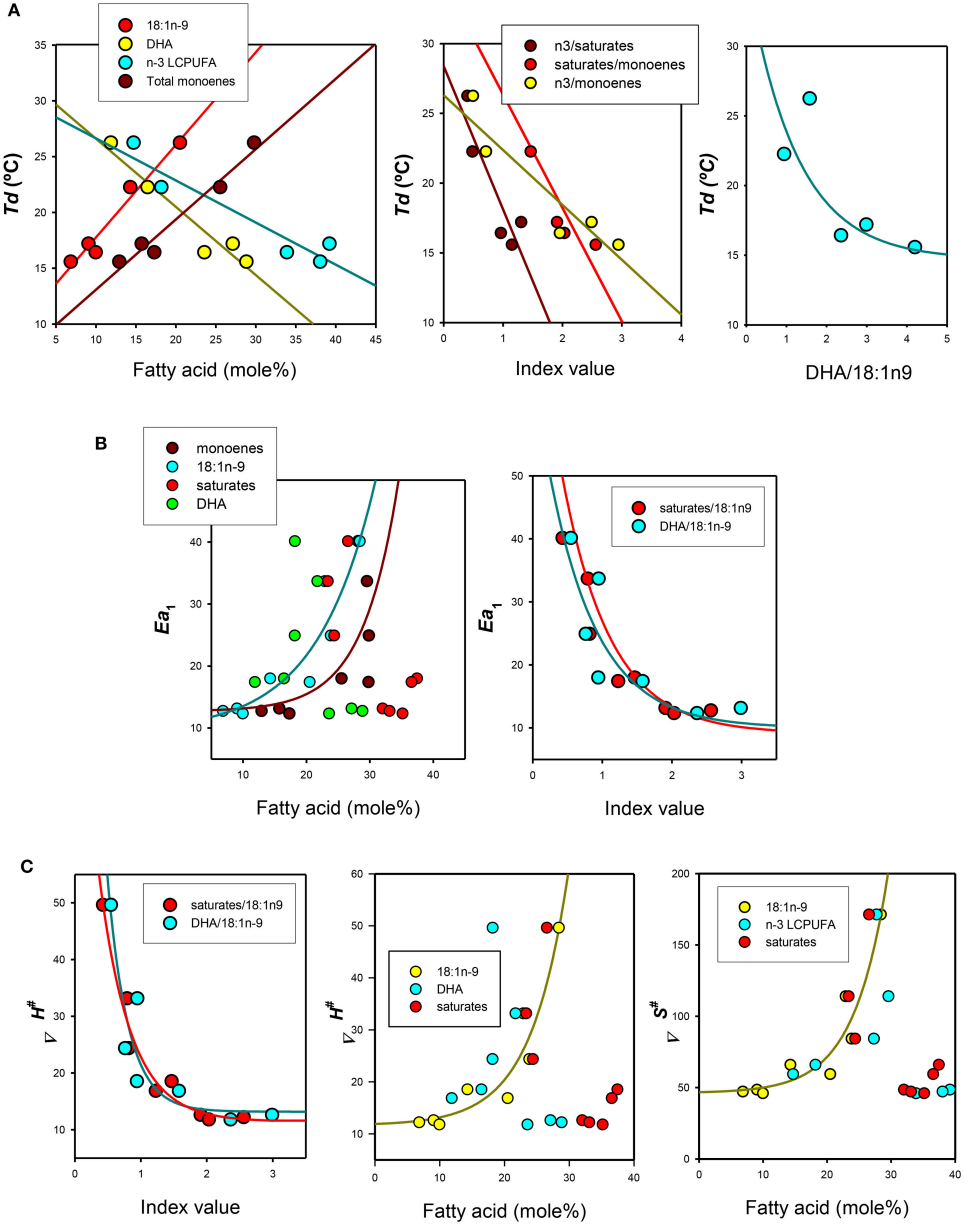
**Relationships between thermodynamic signatures of Na^**+**^-K^**+**^-ATPase from gilthead seabream epithelia and their lipid features. (A)** Effects of individual, grouped or ratios fatty acids on Arrhenius (discontinuity) breakpoints (*Td*). **(B)** Effects of lipid parameters on activation energies below *Td or I*_*t*_*:* (*Ea*_1_). **(C)** Effects of lipid ratios and selected fatty acids on activation enthalpy (Δ*H*^‡^) and activation enthalpy (Δ*S*^‡^*)*.

As n-3 LCPUFA deficiency affected only fatty acid composition of membrane phospholipids, but not lipid classes, levels of most relevant fatty acids were compared with the Arrhenius plot parameters (*Td, Ea*_1_, *Ea*_2_) and activation enthalpy (Δ*H*^‡^) and entropy (Δ*S*^‡^) for enterocytes belonging to the three regions as well as for gill epithelia (Figure 4). Initially, we obtained the Pearson's correlation coefficient matrixes for all fatty acids and ratios which were differentially affected by diets against thermodynamic parameters. First, we found significant relationships between levels of 18:1n-9 (*Td* = 9.46 + 0.83^*^[18:1n-9], *r* = 0.96, *p* < 0.001), total monoenes (*Td* = 6.76 + 0.63^*^[monoenes], *r* = 0.96, *p* < 0.001), DHA (*Td* = 32.72 − 0.61^*^[DHA], *r* = 0.93, *p* < 0.05) or n-3 LCPUFA (*Td* = 3.04 − 0.38^*^[n-3 LCPUFA], *r* = 0.91, *p* < 0.05), and *Td* (Figure 4A, left panel). Noticeably, it was observed an opposite influence of monoenes (positively related) and n-3 polyunsaturated fatty acids (negatively related) and *Td*. The positive effect of monoenes was also related, on one side, to saturated fatty acids as revealed by the negative relationships between saturates/18:1n-9 (*Td* = 34.48 − 8.012^*^[saturates/18:1n-9], *r* = 0.85, *p* < 0.05) and *Td*, and to n-3 LCPUFA, on the other side, as indicated by the negative correlation between n-3 LCPUFA/monoenes (*Td* = 26.30 − 3.93^*^[n-3 LCPUFA/monoenes], *r* = 0.88 *p* < 0.05) and *Td* (Figure 4A, middle panel). More precisely, this later relationship could be defined as a very significant negative exponential relationship between the ratio DHA/18:1n-9 (*Td* = 14.69 + 20.66^*^e^−0.81*[DHA/18:1n-9]^, *r* = 0.96, *p* < 0.005) and *Td* (Figure 4A, right panel). Since *Td* disappeared in n-3 LCPUFA deficient enterocytes (but not in gills) these epithelia were excluded from these regression analyses.

On the other hand, *Ea*_1_ was only related to total monoenes and particularly to 18:1n-9 (*Ea*_1_ = 14.43 + 0.086^*^[1.2^[18:1n-9]^], *r* = 0.91, *p* < 0.005) levels, but not obvious relationships were observed for saturated or polyunsaturated fatty acids individually (Figure 4B, left panel). However, these later two lipid groups appear to substantially affect *Ea*_1,_since their ratios to 18:1n-9 provided good estimations of *Ea*_1_(Figure 4B, right panel),revealing in both cases very significant exponential decays.

Finally, we assessed the relationships between activation enthalpy (Δ*H*^‡^) and activation entropy (Δ*S*^‡^) as dependent variables and lipid parameters. We found that enthalpy of activation was closely related to the DHA/18:1n-9 ratio (Δ*H*^‡^ = 13.17 − 198.7^*^e^−3.17*[DHA/18:1n-9]^, *r* = 0.92, *p* < 0.01) and to saturates/18:1n-9 (Δ*H*^‡^ = 11.43–94.57^*^e^−2.11*[saturates/18:1n-9]^, *r* = 0.98, *p* < 0.001) (Figure 4C, left panel). Enthalpy (Δ*H*^‡^) of activation was also positively (and exponentially) related to monoenes, in particular to 18:1n-9 (Δ*H*^‡^ = 11.63 + 0.28^*^e^−0.17*[/18:1n-9]^, *r* = 0.96, *p* < 0.005) but not to n-3 LCPUFA or DHA (Figure 4C, middle panel). In the case of activation entropy (Δ*S*^‡^), two relevant relationships were observed, i.e., for 18:1n-9 (Δ*S*^‡^ = 46.22 − 0.57^*^e^−0.18^*^[18:1n-9]^, *r* = 0.96, *p* < 0.005) (shown in Figure 4C, right panel), and for the ratio DHA/18:1n-9 ratio (Δ*S*^‡^ = 50.38 + 778.31^*^e^−3.46*[DHA/18:1n-9]^, *r* = 0.92, *p* < 0.01) (not shown).

## Discussion

In the present study, we have assessed the potential involvement of membrane lipid profiles and thermodynamic properties of the Na^+^-K^+^-ATPase in four populations of epithelial cells in the gilthead seabream. First, we observed that fatty acid composition of membrane phospholipids slightly differ between enterocytes isolated from the different intestinal segments. However, most dramatic differences were observed between enterocytes and branchial epithelial cells. To the best of our knowledge this is the first study convincingly demonstrating such heterogeneities in membrane lipid composition of epithelial cells. Highlighting main differences, we observed that, under control conditions, enterocytes are more enriched in long-chain polyunsaturated fatty acids (mainly DHA), total polar lipids, PC, PE, PS, PI, and PG, compared to gill epithelial cells. Conversely, branchial cells contain higher levels of total neutral lipids (mainly CHO, FFA, MAG, and TAG), and 20-carbons polyunsaturated fatty acids (ARA and EPA), as well as monoenes. These features allowed a neat discrimination between epithelial cells in the multivariate analyses performed here, which allowed us to define a differential lipid fingerprint between epithelial cells of intestinal and branchial origins. As the biochemical structure of membrane bilayer is finely tuned to cope with the specific cellular functions, it can be speculated that in gill epithelia, membrane phospholipids are more adequate to provide an efficient permeability transmembrane barrier against severe osmo-ionic gradients (Wood and Shuttleworth, 1995; Evans et al., 2005). Another conclusion that can be extrapolated from these data, is that as membrane surface is greater in enterocytes than in branchial cells (due to the presence of an extensive brush border), it would be expected a higher level of TPL. On the other hand, epithelial branchial cells exhibit higher levels of FFA, MAG, and TAG, which is in consonance with the high density of mitochondria and higher metabolic rates, especially in chloride cells (Wood and Shuttleworth, 1995; Evans et al., 2005), which accomplish the major task of NaCl extrusion against a very large electrochemical gradient.

The second goal of our study was the assessment of the extent to which lipid profiles in epithelial cells might be affected by dietary modifications. Our dietary manipulation consisted in providing an n-3 PUFA-deficient diet which contained similar amount of total lipids compared to control standard diets, but changed proportions of PUFA, monoenoic and saturated fatty acids. The results showed that deficient diets have a profound impact on lipid profiles in enterocytes. With the sole exception of TAG which were significantly increased in all intestinal segments studied, lipid classes remained unaffected. However, most important changes were observed in fatty acid composition of membrane phospholipids. Thus, levels of total monoenes (mainly 18:1n-9) were significantly increased, while DHA, total n-3 LCPUFA and 18:3n-3, were reduced. All these findings revealed a direct influence of diet composition on the enterocyte membrane composition. However, it is worthwhile mentioning that the increase in 18:1n-9 in membrane phospholipids may also be part of a homeoviscous strategy to keep membrane fluidity in conditions of LCPUFA depletion. Strikingly, fatty acid composition of branchial cells from the same animals remained very similar between control and deficient diets, with only small changes in some minor fatty acids. These observations are very relevant from the homeostatic point of view, and pinpoints to the existence of powerful lipostatic mechanisms responsible for the biochemical stability of branchial cell membranes (including chloride cells, mucous cells, epithelial respiratory cells and pillar cells). This indeed leads us to hypothesize that the extremely relevant physiological functions of branchial cells (Wood and Shuttleworth, 1995; Evans et al., 2005) are tightly linked to a precise lipid composition in cell membranes.

The next goal in the present study was the assessment of how changes in lipid profiles of cell membranes might affect the thermodynamic properties of epithelial Na^+^-K^+^-ATPase. Results from Arrhenius plots indicate that the overall reactions of ATP hydrolysis for the Na^+^-K^+^-ATPase activities from all sources followed convex behaviors (Truhlar and Kohen, 2001) with temperature discontinuity points (*Td*) and two activation energies, above (*Ea*_1_) and below (*Ea*_2_) the discontinuity, with *Ea*_1_>*Ea*_2_. Slight differences in these parameters were detected for control enterocytes, but values for control gill epithelia were substantially different, being *Td* and *Ea*_1_ higher than in enterocytes. Similar results have been observed in mucosal scrapings (Almansa et al., 2003) and branchial microsomal fractions from gilthead seabream (Ventrella et al., 1990) and in the sea bass *Dicentrarchus labrax* (Ventrella et al., 1993) reared under similar conditions.

Thermodynamically, the lower *Td* values obtained for enterocytes imply lower values for the thermotropic transitions of protein-lipid and protein-protein interactions and, coherently, a temperature reduction in the activation energies-shift point (Truhlar and Kohen, 2001). Accordingly, the enzyme from pyloric caeca would display lower activation energy above 17.20°C, which would allow an increase in the enzyme activity at lower temperatures (close to *Td*), compared with anterior and posterior enterocytes, whose shift points were clearly lower. Conversely, the enzyme from gills would require substantially higher temperatures (above 22.26°C) to benefit from a lower activation energy (as inferred from *Ea*_2_). This apparent unfavorable condition may not be compensated by the Δ*H*^‡^ values (both above and below Td) found in gill Na^+^-K^+^-ATPase. These assertions have been demonstrated experimentally in enzyme activity assays performed under standard conditions, where branchial Na^+^-K^+^-ATPase specific activity measured at 20°C was found ~50% lower than in averaged enterocytes. Also, these results are in line with lower activation enthalpy for enterocytes compared to gill epithelia (Table 4). Thus, it seems that there exist environmental restrictions leading to restricted degrees of freedom (i.e., translational, rotational, and vibrational) in the transition state for the hydrolysis of ATP by Na^+^-K^+^-ATPase in branchial membranes. This is perhaps the price must be paid in gill epithelia to cope with extreme osmotic and ionic gradients far from equilibrium. It is known that the Na^+^-K^+^-ATPase reaction cycle includes two major conformational intermediates, namely *E1* (with high affinity for ATP) to *E2* (with phosphatase activity) (Schuurmans-Stekhoven and Bonting, 1981; Skou and Esmann, 1992; Kaplan, 2002), and that the transition between them occurs with a large enthalpy and entropy change. Moreover, changes in membrane order (reduction) favors the *E1* conformation and ATP binding (i.e., by PUFA or ethanol) while the increase in membrane order by reduced temperature or PUFA depletion, favors the *E2* conformation with ATP hydrolysis and K^+^ binding (Swann, 1986; Skou and Esmann, 1992; Kaplan, 2002). Thus, the higher absolute values of Δ*H*^‡^ (and Δ*S*^‡^) of activation for the hydrolysis of ATP in gill Na^+^-K^+^-ATPase suggest more ordered membranes, where the stabilization forces required for the transition of *E1* to *E2* conformations for hydrolysis are higher than for enterocytes. Indeed, our analyses of membrane lipid composition (with significantly lower contents of total n-3 LCPUFA in gill epithelia) and the lower membrane unsaturation index found in gill cell membranes (194.35) compared to enterocytes' (ranging 244.4–256.7) support the thermodynamic observations and may well explain the observed differences between intestinal and branchial epithelia.

**Table 4:** **Summary of thermodynamic parameters calculated for epithelial cells from control individuals and animals fed LCPUFA deficient diets**.

	***Ea_1_* (kcal/mol)**	***Ea_2_* (kcal/mol)**	***Td* (°C)**	**Δ*H*^‡^ < *Td* (kcal/mol)**	**Δ*H*^‡^ >*Td* (kcal/mol)**	**Δ*S*^‡^ < *Td* (kcal/°K/mol)**	**Δ*S*^‡^ > *Td* (kcal/°K/mol)**
**CONTROLS**							
Gill	17.98	7.72	22.26	18.53	10.06	65.96	36.87
Pyloric caeca	13.14	5.46	17.20	12.59	4.92	48.50	22.07
Anterior intestine	12.33	6.48	16.42	11.78	7.00	46.01	25.87
Posterior intestine	12.75	8.28	15.58	12.20	7.77	47.13	31.78
**LCPUFA DEFICIENT**							
Gill	17.40	4.40	26.24	16.84	3.83	59.41	15.92
Pyloric caeca	50.13^#^		26.09^#^	49.61^#^		171.27^#^	
Anterior intestine	24.91^#^		27.22^#^	24.36^#^		84.26^#^	
Posterior intestine	33.68^#^		25.77^#^	33.14^#^		113.93^#^	

# *Values were obtained below I_t_*.

The relationships between epithelial membrane lipids and thermodynamic properties of Na^+^-K^+^-ATPase was further demonstrated in the n-3 PUFA deficiency experiments. This maneuver dramatically affected lipid profiles in enterocytes. As mentioned before, the main effects being a considerable increase in oleic acid and a substantial DHA depletion. Paralleling these findings, *Td* disappeared and *Ea*_1_ increased by 2–3.8-fold compared to control animals. LCPUFA depletions also augmented the thermosensitivity of intestinal Na^+^-K^+^-ATPase and reduced the values for inactivation temperature (*I*_*t*_). Noticeably, none of these parameters were significantly affected in gill epithelia, which reinforce the hypothesis that branchial cells are endowed with a highly efficient lipostatic mechanism ensuring the biochemical stability of membrane lipids (and lipid-lipid and lipid-protein interactions), ultimately giving support to the splendid physiological complexity of fish gills.

Severe changes in absolute values of Δ*H*^‡^ (and Δ*S*^‡^) of activation for the hydrolysis of ATP were observed in enterocytes (but again not in gill epithelia), which increased by 2–4-fold for Δ*H*^‡^ and 1.8–3.5 fold for Δ*S*^‡^ below inactivation temperature (*I*_*t*_). These observations suggest more ordered membranes and higher stabilization forces for the transition *E1* to *E2* conformations. In agreement, unsaturation indexes in enterocytes (but not in branchial cells) were reduced by LCPUFA depletion by 14% (index values ranging 261-227 in controls, and 206-227 in LCPUFA deficient cells). The deleterious effects of increased *Ea*_1_, Δ*H*^‡^ and Δ*S*^‡^ were readily demonstrated in the specific activity assays (as measured at 20°C, the rearing temperature of seabream). Indeed, unlike branchial preparations, in enterocytes' homogenates, Na^+^-K^+^-ATPase specific activity fell by average 82.8% compared to controls.

In order to explore more deeply the individual relationships between specific membrane lipids and lipid ratios, as independent variables, and thermodynamic parameters, we used non-linear regression analyses. We found that *Td* was significantly negatively related to DHA and positively to 18:1n-9 (and monoenes). Expectedly, the ratio n-3 LCPUFA/monoenes (and more precisely the ratio DHA/18:1n-9) was negatively related to both *Td*, following a negative exponential relationship. Overall, these data indicates that the relative amounts of 18:1n9 and DHA in membrane phospholipids are critical in determining the discontinuity point and the transition *Ea*_1_↔*Ea*_2_ and pinpointed that small changes in DHA and/or 18:1n-9, have a great impact in setting *Td* as per their exponential relationship. Moreover, the same ratio (DHA/18:1n-9) was also found to be a significant predictor of *Ea*_1_ and Δ*H*^‡^ values, observing in both cases negative exponential equations describing their relationships. In these cases, both total monoenes and 18:1n-9 (but not saturates or DHA alone) were significantly related to *Ea*_1_, Δ*H*^‡^ and Δ*S*^‡^ but following exponential relationships, which indicates a very relevant role of 18:1n-9. Overall, these data indicate the relevant effects of DHA and monoenoic fatty acids (and their relative proportions) in setting thermodynamic traits for Na^+^-K^+^-ATPase, at least for ATP hydrolysis.

Finally, current available information based on reconstitution and crystallographic studies may provide, at least in part, a molecular interpretation in support of our observations. Thus, recent studies using purified detergent-soluble recombinant α/β or α/β/FXYD Na^+^-K^+^-ATPase complexes, have revealed three separate functional effects of phospholipids and cholesterol that are exerted at separate binding sites for phophatidylserine/cholesterol (site A), polyunsaturated phosphatidylethanolamine (site B), and saturated PC or sphingomyelin/cholesterol (site C) (Cornelius et al., 2015; Habeck et al., 2015). These binding sites likely correspond to three lipid-binding pockets identified in the crystal structures of the Na^+^-K^+^-ATPase (Shinoda et al., 2009; Laursen et al., 2013). The three sites appear to have different effects on Na^+^-K^+^-ATPase complexes, being site A stabilizing, site B stimulatory and site C inhibitory (Cornelius et al., 2015; Habeck et al., 2015). Therefore, it is expected that direct and specific interactions of different phospholipids and cholesterol within the protein molecular framework, will determine both the stability and molecular activity of Na^+^-K^+^-ATPase, eventually giving membrane lipid composition an essential role in its physiological regulation. As it has been mentioned before, the activating site is associated to polyunsaturated fatty acid binding, especially when esterifying the *sn*-2 position of neutral lipids such phosphatidylethanolamine or phosphatidylcholine. Indeed, these two phospholipids represent by average 66% of epithelial TPL (Tables 2, 3), and DHA is, by far, the most abundant fatty acid in epithelial polar lipids. Recently, the stimulation of Na^+^-K^+^-ATPase activity of the purified human α1β1 or α1β1FXYD1 complexes by neutral PUFA PC or PE has been reported, being the stimulation structurally selective for neutral phospholipids (Haviv et al., 2013). The structural selectivity for the neutral phospholipid and asymmetric saturated plus PUFA fatty acyl chain structure is actually a strong indication for a specific interaction with Na^+^-K^+^-ATPase. Indeed, molecular modeling of *E1*~*P* and *E2*·*P* conformations bound to any of these polyunsaturated phospholipids suggests that their specific binding may facilitate the *E1P*↔*E2P* conformational transition (mainly by lowering the activation energy) (Cornelius et al., 2015). In addition, DHA (and other LCPUFA) impose a general physical effect on the bilayer physicochemical state as it has conformational properties that keep highly structured but fluid membrane bilayer capable to accommodate rapid protein conformational changes (Rabinovich and Ripatti, 1991; Stillwell and Wassall, 2003; Diaz et al., 2012). Presumably, adjustment of DHA levels within membrane phospholipids (mainly phosphoglycerides) would accomplish effective physicochemical changes over a wide temperature range, ensuring the adaptation of cell membranes to environmental fluctuations and metabolic activity (Sargent et al., 1995). The latter being considered an important mechanism involved in thermal acclimation in ectothermic organisms (Raynard and Cossins, 1991; Else and Wu, 1999; Ernst et al., 2016). Interestingly, the stimulatory effects of polyunsaturated PE or PC seem to be independent of cholesterol and the FXYD protein (Cornelius et al., 2015). This is relevant because cholesterol is quite abundant in epithelial lipid profiles (the second most abundant neutral lipid after TAG, and accounting for ~40% of TNL), and because cholesterol has been long recognized to be essential for optimal Na^+^-K^+^-ATPase activity (Crockett and Hazel, 1997; Cornelius, 2001; Cornelius et al., 2015). The effects of cholesterol on Na^+^-K^+^-ATPase are associated to its ability to impose a high degree of conformational order on the phospholipids acyl chains thereby stabilizing the liquid-ordered lipid phase, which is dominated by changes in activation entropy (Cornelius et al., 2015), but also to direct interaction with site A (Shinoda et al., 2009; Habeck et al., 2015). However, as we observed no changes in cholesterol contents, neither between tissues nor between diets (Table 3), observed differences in activation Δ*H*^‡^ and Δ*S*^‡^ cannot be explained by distinct membrane cholesterol.

Finally, we wish to introduce a word of caution on the extent to which our results may be interpreted. Indeed, although we have empirically studied the thermodynamic parameters of the epithelial Na^+^-K^+^-ATPase from intestinal and branchial origins, and correlated them with the membrane phospholipid composition, it is not possible to provide molecular details on the real enzyme-membrane interactions nor giving precise insights into the physical mechanisms underlying the observed responses.

In conclusion, the results illustrated in the present study reveal that subtle differences in the lipid matrix of the lipid microenvironment surrounding the Na^+^-K^+^-ATPase may explain the heterogeneous thermodynamic features of the Na^+^-K^+^-ATPase in epithelia from the gilthead seabream reared under control conditions. In enterocyte preparations, these effects were exacerbated by induction of membrane n-3 LCPUFA deficiency. Noteworthy, epithelial cells from gill origin were notably resistant to diet-induced modifications of membrane lipid composition and, consequently, to alterations in the thermodynamic features of the Na^+^-K^+^-ATPase. We conclude that n-3 LCPUFA and 18:1n-9 (together with cholesterol) are critical elements for the fine tuning of the Na^+^-K^+^-ATPase activity within the context of the epithelial plasma membrane, which underlies the essential role of the Na^+^-K^+^-ATPase in the physiological regulation of osmo/ionoregulatory tasks in euryhaline teleost fish.

## Author contributions

RD and CR performed the lipid analyses and specific ATPase activity experiments. TG was in charge of temperature-dependence experiments. MD designed the study, analyzed the data (together with RD) and wrote the manuscript. All authors reviewed the drafted manuscript.

### Conflict of interest statement

The authors declare that the research was conducted in the absence of any commercial or financial relationships that could be construed as a potential conflict of interest.

## References

[B1] Almansa E. Sanchez J. J. Cozzi S. Casariego M. Cejas J. Díaz M. (2001). Segmental heterogeneity in the biochemical properties of the Na^+^-K^+^-ATPase along the intestine of the gilthead seabream (*Sparus aurata* L.). J. Comp. Physiol. B Biochem. Syst. Environ. Physiol. 171, 557–567. 10.1007/s003600100206 11686614

[B2] Almansa E. Sánchez J. J. Cozzi S. Rodríguez C. Díaz M. (2003). Temperature-activity relationship for the intestinal Na^+^-K^+^-ATPase of *Sparus aurata*. A role for the phospholipid microenvironment? J. Comp. Physiol. B 173, 231–237. 10.1007/s00360-003-0327-y 12743726

[B3] Arora A. Esmann M. Marsh D. (1998). Selectivity of lipid-protein interactions with trypsinized Na, K-ATPase studied by spin-label EPR. Biochim. Biophys. Acta 1371, 163–167. 10.1016/S0005-2736(98)00030-3 9630603

[B4] Bell M. V. Henderson R. J. Sargent J. R. (1986). The role of polyunsaturated fatty acids in fish. Comp. Biochem. Physiol. B 83, 711–719. 10.1016/0305-0491(86)90135-5 3519065

[B5] Bogdanov M. Mileykovskaya E. Dowhan W. (2008). Lipids in the assembly of membrane proteins and organization of protein supercomplexes: implications for lipid-linked disorders. Subcell. Biochem. 49, 197–239. 10.1007/978-1-4020-8831-5_8 18751913 PMC2579957

[B6] Brasitus T. A. (1983). Protein-lipid interactions and lipid dynamics in rat enterocyte plasma membranes, in Intestinal Transport, eds Gillies-Baillen M. Gilles R. (New York, NY: Springer-Verlag), 188–197.

[B7] Christie W. W. (1982). Lipid Analysis. Oxford: Pergamon Press.

[B8] Cohen E. Goldshleger R. Shainskaya A. Tal D. M. Ebel C. le Maire M.. (2005). Purification of Na^+^, K^+^-ATPase expressed in *Pichia pastoris* reveals an essential role of phospholipid-protein interactions. J. Biol. Chem. 280, 16610–16618. 10.1074/jbc.M414290200 15708860

[B9] Cornelius F. (2001). Modulation of Na,K-ATPase and Na-ATPase activity by phospholipids and cholesterol. I. Steady-state kinetics. Biochemistry 40, 8842–8851. 10.1021/bi010541g 11467945

[B10] Cornelius F. Habeck M. Kanai R. Toyoshima C. Karlish S. J. (2015). General and specific lipid-protein interactions in Na,K-ATPase. Biochim. Biophys. Acta 1848, 1729–1743. 10.1016/j.bbamem.2015.03.012 25791351

[B11] Cornelius F. Skou J. C. (1984). Reconstitution of (Na^+^, K^+^)-ATPase into phospholipid vesicles with full recovery of its specific activity. Biochim. Biophys. Acta 772, 357–373. 10.1016/0005-2736(84)90153-6 6326830

[B12] Crockett E. L. Hazel J. R. (1997). Cholesterol affects physical properties and (Na^+^, K^+^)-ATPase in basolateral membranes of renal and intestinal epithelia from thermally acclimated rainbow trout. J. Comp. Physiol. B 167, 344–351. 10.1007/s003600050083

[B13] Díaz M. Cozzi S. Almansa E. Casariego M. Bolaños A. Cejas J. (1998). Characterization of intestinal Na^+^-K^+^-ATPase in the gilthead seabream (*Sparus aurata* L.). Evidence for a tissue-specific heterogeneity. Comp. Biochem. Physiol. B 121, 65–76. 10.1016/S0305-0491(98)10052-4

[B14] Díaz M. Marín R. (2013). Brain polyunsaturated lipids and neurodegenerative diseases, in Nutraceuticals and Functional Foods: Natural Remedy, eds Brar S. K. Kaur S. Dhillon G. S. (New York, NY: Nova Science Publishers Inc.), 387–412.

[B15] Diaz M. L. Fabelo N. Marín R. (2012). Genotype-induced changes in biophysical properties of frontal cortex lipid raft from APP/PS1 transgenic mice. Front. Physiol. 3:454. 10.3389/fphys.2012.00454 23205014 PMC3506919

[B16] Dópido R. Rodríguez C. Gómez T. Acosta N. G. Díaz M. (2004). Isolation and characterization of enterocytes along the intestinal tract of the gilthead seabream (*Sparus aurata* L.). Comp. Biochem. Physiol. A 139, 21–31. 10.1016/j.cbpb.2004.06.013 15471677

[B17] Dowhan W. (1997). Molecular basis for membrane phospholipid diversity: why are there so many lipids? Annu. Rev. Biochem. 66, 199–232. 10.1146/annurev.biochem.66.1.199 9242906

[B18] Else P. L. Wu B. J. (1999). What role for membranes in determining the higher sodium pump molecular activity of mammals compared to ectotherms? J. Comp. Physiol. B 169, 296–302. 10.1007/s003600050224 10466220

[B19] Ernst R. Ejsing C. S. Antonny B. (2016). Homeoviscous adaptation and the regulation of membrane lipids. J. Mol. Biol. 428, 4776–4791. 10.1016/j.jmb.2016.08.013 27534816

[B20] Esmann M. Marsh D. (2006). Lipid-protein interactions with the Na, K-ATPase. Chem. Phys. Lipids 141, 94–104. 10.1016/j.chemphyslip.2006.02.018 16580658

[B21] Evans D. H. (1998). The Physiology of Fishes. Boca Raton, FL: CRC Press.

[B22] Evans D. H. Piermarini P. M. Choe K. P. (2005). The multifunctional fish gill: dominant site of gas exchange, osmoregulation, acid-base regulation, and excretion of nitrogenous waste. Physiol. Rev. 85, 97–177. 10.1152/physrev.00050.2003 15618479

[B23] Forbush B. III. (1983). Assay of Na^+^-K^+^-ATPase in plasma membrane preparations: increasing the permeability of membrane vesicles using sodium dodecyl sulfate buffered with bovine serum albumin. Anal. Biochem. 128, 159–163. 10.1016/0003-2697(83)90356-1 6303151

[B24] Gerbi A. Maixent J. M. Barbey O. Jamme I. Pierlovisi M. Coste T.. (1998). Alterations of Na,K-ATPase isoenzymes in the rat diabetic neuropathy: protective effect of dietary supplementation with n-3 fatty acids. J. Neurochem. 71, 732–740. 10.1046/j.1471-4159.1998.71020732.x 9681464

[B25] Gerbi A. Zerouga M. Debray M. Durand G. Chanez C. Bourre J. M. (1993). Effect of dietary α-linolenic acid on functional characteristic of Na^+^/K^+^-ATPase isoenzymes in whole brain membranes of weaned rats. Biochim. Biophys. Acta 1165, 192–198. 10.1016/0005-2760(93)90139-Z 8380337

[B26] Gerbi A. Zerouga M. Debray M. Durand G. Chanez C. Bourre J. M. (1994). Effect of fish oil diet on fatty acid composition of phospholipids of brain membranes and on kinetic properties of Na^+^-K^+^-ATPase isoenzymes of weaned and adult rats. J. Neurochem. 62, 1560–1569. 10.1046/j.1471-4159.1994.62041560.x 8133284

[B27] Habeck M. Haviv H. Katz A. Kapri-Pardes E. Ayciriex S. Shevchenko A.. (2015). Stimulation, inhibition, or stabilization of Na,K-ATPase caused by specific lipid interactions at distinct sites. J. Biol. Chem. 290, 4829–4842. 10.1074/jbc.M114.611384 25533463 PMC4335223

[B28] Haviv H. Habeck M. Kanai R. Toyoshima C. Karlish S. J. (2013). Neutral phospholipids stimulate Na,K-ATPase activity: a specific lipid-protein interaction. J. Biol. Chem. 288, 10073–10081. 10.1074/jbc.M112.446997 23430748 PMC3617245

[B29] Kaplan J. H. (2002). Biochemistry of Na,K-ATPase. Annu. Rev. Biochem. 71, 511–535. 10.1146/annurev.biochem.71.102201.141218 12045105

[B30] Klingenberg H. (1975). Alterations in phospholipid dependent (Na^+^+K^+^)-ATPase activity due to lipid fluidity. Effect of cholesterol and Mg^2+^. Biochim. Biophys. Acta 413, 143–156. 10.1016/0005-2736(75)90065-6 90

[B31] Laursen M. Yatime L. Nissen P. Fedosova N. U. (2013). Crystal structure of the high-affinity Na^+^K^+^-ATPase-ouabain complex with Mg^2+^ bound in the cation binding site. Proc. Natl. Acad. Sci. U.S.A. 110, 10958–10963. 10.1073/pnas.1222308110 23776223 PMC3704003

[B32] Martín V. Almansa E. Fabelo N. Díaz M. (2006). Selective enrichment in polyunsaturated fatty acids in phospholipids from neuronal-derived cell lines. J. Neurosci. Methods 153, 230–238. 10.1016/j.jneumeth.2005.10.019 16337275

[B33] Matsunari H. Hashimoto H. Oda K. Masuda Y. Imaizumi H. Teruya K. (2013). Effects of docosahexaenoic acid on growth, survival and swim bladder inflation of larval amberjack *Seriola dumerili*, Risso. Aquac. Res. 44, 1696–1705. 10.1111/j.1365-2109.2012.03174.x

[B34] Muriana F. J. Ruiz-Gutierrez V. Vazquez C. M. (1992). Influence of dietary cholesterol on polyunsaturated fatty acid composition, fluidity and membrane-bound enzymes in liver microsomes of rats fed olive and fish oil. Biochimie 74, 551–556. 10.1016/0300-9084(92)90153-6 1520734

[B35] Olsen R. E. Henderson R. J. (1989). The rapid analysis of neutral and polar marine lipids using double-development HPTLC and scanning densitometry. J. Exp. Mar. Biol. Ecol. 129, 189–197. 10.1016/0022-0981(89)90056-7

[B36] Rabinovich A. L. Ripatti P. O. (1991). On the conformational, physical properties and functions of polyunsaturated acyl chains. Biochem. Biophys. Acta 1085, 53–62. 10.1016/0022-0981(89)90056-7 1892878

[B37] Raynard R. S. Cossins A. R. (1991). Homeoviscous adaptation and thermal compensation of sodium pump of trout erythrocytes. Am. J. Physiol. 260, R916–R924. 2035703 10.1152/ajpregu.1991.260.5.R916

[B38] Russo G. L. (2009). Dietary n-6 and n-3 polyunsaturated fatty acids: from biochemistry to clinical implications in cardiovascular prevention. Biochem. Pharmacol. 77, 937–946. 10.1016/j.bcp.2008.10.020 19022225

[B39] Sargent J. R. Bell M. V. Bell J. G. Henderson R. J. Tocher D. R. (1995). Origins and functions of n-3 polyunsaturated fatty acids in marine organisms, in Phospholipids: Characterization, Metabolism and Novel Biological Applications, eds Ceve G. Paltauf F. (Champaign, IL: American Oil Chemical Society Press), 248–259.

[B40] Schuurmans-Stekhoven F. Bonting S. L. (1981). Transport adenosine triphosphatases: properties and functions. Physiol. Rev. 61, 1–76. 6258180 10.1152/physrev.1981.61.1.1

[B41] Shinoda T. Ogawa H. Cornelius F. Toyoshima C. (2009). Crystal structure of the sodium-potassium pump at 2.4. A resolution. Nature 459, 446–450. 10.1038/nature07939 19458722

[B42] Skou J. C. Esmann M. (1992). The Na-K-ATPase. J. Bioenerg. Biomembr. 24, 249–261. 1328174 10.1007/BF00768846

[B43] Spector A. A. Kim H. Y. (2015). Discovery of essential fatty acids. J. Lipid Res. 56, 11–21. 10.1194/jlr.R055095 25339684 PMC4274059

[B44] Stillwell W. Wassall S. R. (2003). Docosahexaenoic acid: membrane properties of a unique fatty acid. Chem. Phys. Lipids 126, 1–27. 10.1016/S0009-3084(03)00101-4 14580707

[B45] Swann A. C. (1986). Brain Na^+^,K^+^-ATPase: alteration of ligand affinities and conformation by chronic ethanol and noradrenergic stimulation *in vivo*. J. Neurochem. 47, 707–714. 10.1111/j.1471-4159.1986.tb00669.x 3016182

[B46] Truhlar D. Kohen A. (2001). Convex Arrhenius plots and their interpretation. Proc. Natl. Acad. Sci. U.S.A. 98, 848–851. 10.1073/pnas.98.3.848 11158559 PMC14672

[B47] Ventrella V. Pagliarani A. Pirini M. Trigari G. Trombetti F. Borgatti A. R. (1993). Lipid composition and microsomal ATPase activities in gills and kidneys of warm- and cold-acclimated sea bass (*Dicentrarchus labrax* L.). Fish. Physiol. Biochem. 12, 293–304. 10.1007/BF00004414 24202871

[B48] Ventrella V. Trombetti F. Pagliarini A. Trigari G. Borgatti A. R. (1990). Gill (Na^+^+ K^+^)- and Na^+^-stimulated Mg^2+^-dependent ATPase activities in the gilthead bream (*Sparus auratus* L.). Comp. Biochem. Physiol. B 95, 95–105. 10.1016/0305-0491(90)90254-q 2158872

[B49] Wheeler K. P. Whittam R. (1970). The involvement of phosphatidylserine in adenosine triphosphatase activity of the sodium pump. J. Physiol. 207, 303–328. 10.1113/jphysiol.1970.sp009063 4250771 PMC1348708

[B50] Wood C. M. Shuttleworth T. J. (1995). Cellular and Molecular Approaches to Fish Ionic Regulation. San Diego, CA: Academic Press, Inc.

[B51] Yeagle P. L. Young J. Rice D. (1988). Effects of cholesterol on (Na^+^, K^+^)-ATPase ATP hydrolyzing activity in bovine kidney. Biochemistry 27, 6449–6452. 10.1021/bi00417a037 2851324

